# Beliefs and intention of heterosexual couples about undertaking Couple’s HIV Testing and Counselling (CHTC) services in Ethiopia

**DOI:** 10.1186/s12913-020-4947-7

**Published:** 2020-02-05

**Authors:** Tewodros Getachew Hailemariam, Patrick Rawstorne, Mitike Molla Sisay, Sally Nathan

**Affiliations:** 1 School of Public Health & Community Medicine, University of New South Wales, Sydney, Australia; 2 School of Public Health, Wolaita Sodo University, Wolaita Sodo, Ethiopia; 3 School of Public Health, Addis Ababa University, Addis Ababa, Ethiopia

**Keywords:** Ethiopia, Heterosexual, Couples, HIV, Testing, CHTC

## Abstract

**Background:**

Couples HIV Testing & Counselling (CHTC) service is an approach that may enable more people to be reached and tested for HIV. However, little is known about how couples may use this service and what they think about CHTC as an approach to finding out their HIV status. This study aimed to understand how individuals who had ever been in an ongoing heterosexual relationship for 6 months or more intended to use CHTC in Ethiopia and their beliefs about its benefits and potential harms.

**Methods:**

Qualitative in-depth interviews were conducted in Addis Ababa, the capital city of Ethiopia, in 2017. Semi-structured interviews were undertaken with individuals who had ever been in an ongoing heterosexual relationship (*n* = 21) and key-informants (*n* = 11) including religious leaders, health care providers, and case managers. The interviews were transcribed verbatim, and an inductive thematic analysis was conducted. The data were coded to look for concepts and patterns across the interviews and relevant themes identified which captured key aspects related to the individual’s views on undertaking HIV testing with a sexual partner.

**Results:**

Most participants regarded CHTC as an important HIV testing approach for people who are in an ongoing heterosexual relationship and expressed the view that there was “nothing like testing together”. However, many of the individual participants revealed they would prefer first to get tested alone to find out their own HIV status. They feared the consequences if they were HIV-positive, including accusations of infidelity, relationship break-up, and being exposed in the community. Many also reported being pressured to undertake CHTC before marriage by a third party, including religious institutions. Key informant interviews also discussed the requirements for CHTC before marriage.

**Conclusion:**

The findings of this study suggest that people may be concerned about undertaking couples HIV testing without prior individual HIV testing. The intention of many to first test alone has policy and cost implications and underscores the possible harms of the implementation of CHTC in Ethiopia. Future research should examine whether the views identified in this qualitative study are reflected more broadly among couples in the community.

## Introduction

HIV Testing and Counselling (HTC) is an essential entry point to initiate treatment, care and support to prevent further transmission [1, 2]. The World Health Organisation (WHO) has issued guidelines and standards to implement HTC services worldwide [1, 3–5]. Both the guidelines and HIV testing approaches have evolved over time in response to social and political changes, and advancements in HIV testing technology [6].

Couples HIV Testing and Counselling (CHTC) is one approach being promoted by the WHO as a way of targeting people who are in, or planning to be in an ongoing sexual relationship on regular basis. CHTC involves both members of a couple receiving pre-and post-test counselling, and test results together [3]. Despite increasing interest in understanding the contribution of different HTC approaches to increasing the uptake rate of HIV testing [7, 8], limited attempts have been made to understand each approach and their strengths and limitations. In Sub-Saharan Africa (SSA), where more than 70% of People Living with HIV (PLHIV) live, a large body of epidemiological research has been focused on how CHTC could increase uptake of HIV testing [9–11]. However, HIV is a sensitive issue in the socio-cultural context of SSA, as it is in many other places, and individuals could be exposed to unintended psycho-social impacts following CHTC [11, 12]. Awareness of some of these consequences may partly explain why the number of couples choosing CHTC has been relatively low compared with expectations in the region [13].

Reasons why couples are not using CHTC services have been the focus of recent qualitative studies. For instance, studies [14–18] in Uganda have shown that mistrust in marriage or partnerships, fear of consequences, and conflicts with work schedules were stated barriers to undertaking CHTC among married couples. Similarly, another study [19] in Tanzania reported that power imbalances between men and women, and male-unfriendly healthcare settings during antenatal care were barriers to undertaking CHTC among pregnant women and their male partners in Tanzania. It has also been argued that CHTC may help individuals who are in HIV-discordant (mixed HIV status) relationships facilitate mutual disclosure and the initiation of ART for the person who is diagnosed HIV-positive [20–22]. However, none of these studies explored couples’ intentions and beliefs around CHTC in the general community.

Previous studies on CHTC that have been conducted within the context of pregnancy and Prevention of Mother to Child Transmission (PMTCT) are different to the current study, as the health of unborn children is a priority for expecting parents in this context. In Ethiopia like most SSA countries, where couples are considered a priority group for increasing uptake of HIV testing services, understanding service users and stakeholder perceptions and perspectives on CHTC in the general community is important. This study, therefore, aimed to explore people’s beliefs about undertaking HIV testing with a sexual partner including couples outside of the PMTCT environment, and whether and how they intended to use this type of HIV testing approach in the Ethiopian context. It is anticipated that the findings of this study may help to inform HIV testing policies and programs in Ethiopia and other countries that promote CHTC.

## Methods

### Study design and setting

A qualitative interview study was conducted from September–December 2017 in Addis Ababa, Ethiopia. Interviews were undertaken in two hospitals, and at residential places or offices as requested by participants. The two hospitals were Zewditu Memorial and Yekatit-12 Memorial which are general hospitals located in two sub-cities of Addis Ababa namely Lideta and Arada. These two hospitals are administered by the city not federally and are therefore used by residents of the city primarily versus those from regional areas who are more likely to attend the other hospitals. Zewditu Memorial is also the first hospital in Ethiopia to offer ART to people living with HIV.

Addis Ababa is the capital city of Ethiopia with a population of more than 3.2 million people [23]. In 2018, the national Demographic and Health Survey report showed that the city had the second highest-level of HIV prevalence of any city or regional state in Ethiopia with an estimated HIV-prevalence of 3.4% of the adult population aged 15–49 [24].

### Participants and recruitment

Study participants were recruited through purposive sampling [25] using two recruitment strategies: 1) invitation via health care provider upon arrival at one of the follolwing clinics: Antiretroviral therapy (ART), Prevention Mother-to-child-transmission (PMTCT), Tuberculosis (TB), or Antenatal Care (ANC) clinics in the two hospitals (Zewiditu Memorial and Yekatit-12 hospitals), and 2) a generic letter inviting individuals who were eligible for the study through the participants who had already participated. The individuals who took part were asked to pass on this letter to other potential participants, creating a snowball sample. The decision to use both purposive and snowball sampling was made to extend recruitment to other participants in residential and work settings so as to maximise the diversity of the study participants involved.

Participants that were eligible for this study were individuals who had ever been in an ongoing heterosexual relationship for 6 months and more, and were 18 years of age or older. The intention of the research was to include a diverse profile of participants including those who had ever experienced having an HIV test either alone or with their sexual partner/s; individuals who had never been tested before; and individuals from different types of relationships (cohabiting, married, divorced, remarried, and single).

Healthcare providers at the two hospitals provided a verbal explanation which was a summary of key elements of the research using the participant information statement approved for the study, to the potential participants. Those participants who were interested in taking part were requested to meet with the field research team (i.e. the first author and/or one female research assistant) who were at the hospital for data collection. A member of the field research team then provided more detailed information about the study, after which those who were interested in taking part provided full informed consent.

Inviting potential participants through participants who had already consented and completed an interview facilitated further recruitment of individuals from residential areas and workplaces in the city who may not have recently attended the hospital. Those individuals who travelled for the purpose of the interview were provided 50birr = 2USD for transport reimbursement. The key-informants were people who had rich experiences related to couple’s issues such as HIV testing, marriage and family counselling. They were recruited using purposive sampling which enabled targeted recruitment of participants to those people who were most likely to provide useful data to help inform answers to the research question [25]. Key informants (KI) were recruited via their respective institutions and the first author approached selected health facilities and religious institutions to identify the key-informants who were directly involved in CHTC. The first author explained the key aspects of the study to the key-informants. Those who were willing to participate had an interview time scheduled. The individuals that were regarded as key-informants for this study included religious leaders and health care providers who worked closely with couples seeking to marry and in relation to testing for HIV. The key-informants were also informed about the research and those who were interested in participating provided their consent to do so.

### Data collection and instrument

Semi-structured in-depth interviews were conducted with individuals and key-informants (See Additional files 1 and 2). An interview guide was drafted and developed in reference to relevant existing literature on salient beliefs and theory of planned behaviour [26–28] and discussion among the field research team. The interview guide was pre-tested with five individuals who were in an ongoing heterosexual relationship to check the questions were clear and easy to understand. Minor wording and probes were adjusted following the pre-test and as interviews progressed and key issues identified in earlier interviews included in subsequent interviews [29]. The interview guide consisted of questions and probes for the following categories: intention and attitude towards CHTC (e.g. What do you think are the advantages and disadvantages of CHTC?); perceived social pressure to undertake CHTC (e.g. Are there any individuals or groups who would approve or disapprove of your intention undertake CHTC or not?); how easy or difficult would it be to undertake CHTC?; and would your preference be to undertake HIV Testing and Counselling (HTC) as an individual or as a couple (e.g. What would be a preferred way of HIV testing service for individuals who are in an ongoing sexual relationship?).

The first author and an experienced female research assistant (RA), both fluent in using the local language (Amharic), collected the data. Before starting data collection, the first author provided training to the research RA on the overall research objectives, contents of the interview guide and probing techniques to be used. Approaches to interviewing in this context were also discussed and regular sharing of experiences of the interviews undertaken to maximise data quality and rapport. The first author interviewed most of the male participants and the female RA interviewed most of the female participants. All interviews with key-informants were conducted by the first author. A digital recording was used for all interviews which lasted between 20 and 60 min.

### Data analysis

All interview audio files were transcribed verbatim in English by the first author who is proficient in both Amharic and English languages and imported into NVivo 11, a data management tool, for analysis. A thematic analysis was done in multiple steps with the first step focusing on familiarisation with the data [29, 30]. The transcripts were first read and re-read with taking notes about patterns and important concepts. The transcripts were then coded looking in a systematic way for key concepts and patterns of meaning across the entire dataset. Generating initial codes, which was the second stage of the thematic analysis approach taken, produced a long list of codes. In the next step, the list of codes served as the foundation to find agreement on candidate themes which were latter refined into core themes which are presented in this paper. As detailed by Braun & Clarke [30] and Terry et al. [29] the authors together reviewed potential themes to ensure they captured key concepts in the data. The core themes and sub-themes were checked against the research questions to ensure they provided a clear and coherent account of participants’ perspectives and answered the research questions. The core themes and sub-themes are illustrated with quotes from respondents in this paper.

### Rigor and reflexivity

To ensure rigor in the current study, there were a number of key strategies employed. These strategies included interviewing participants with diverse profiles (marital status, age, gender), interviewing key informants in addition to clients, involving multiple authors in themes development, and transparency about the conduct of the research. The initial stage of thematic analysis was conducted by first author (TGH). A triangulation (an agreement on interpretations of data) in agreeing themes was undertaken through multiple discussion among the authors [29]. Moreover, we followed Yardley [31] recommendations for transparency of methods in a qualitative study. As such, in this study, transparency is reflected in the detail in how the data were collected, analysed, and the role of the research team in the interpretation of data.

An awareness of researcher as an instrument within the research process is important in qualitative research. It is therefore important to provide details about the researcher(s), the setting, context and social phenomenon to aid transferability [32, 33]. The field research team (i.e. the first author and the research assistant) who completed the interviews had direct experiences of CHTC. Both members of the field research team had gone through CHTC at the request of either a religious institution or family before getting married. In these ways, the field research team had an understanding of the phenomenon and the cultural context in which CHTC occurs. These two people individual experiences of CHTC is likely to have influenced their views about CHTC which was something that required ongoing reflection, particularly during analysis and interpretation. The experience of undertaking CHTC, being Ethiopian and not being a healthcare employee, often helped build rapport with participants. However, both the first author and the research assistant were careful not to disclose their experiences in detail and influence the participant to only provide a more socially desirable response.

It’s also important to note that some of the interviews took place within health facilities without a prior appointment for the interview. Interviews were conducted only after the health services appointment was completed. The interviewers both explained that they were not employed by the health service and that the interview was confidential. However, the setting of a health service could influence how and in what ways participants shared about their views and experiences.

Although participants were asked if they preferred a certain gender of interviewer, most did not state a preference. Those that prefered a specific gender interviewer were able to be interviewed accordingly. The role of gender in the interviews is important to consider due to the sensitive nature of the topic of interview. The field researchers paid attention to the dynamics in the interview and worked to address any issues that impacted rapport as they arose.

Some interviews were completed by the female research assistant, who had a master’s degree in public health. More than half of the interviews were conducted by the first author who is an Ethiopian male undertaking a PhD degree in Australia. The education level of the two interviewers was often higher than that of some participants and may have created a power imbalance. To overcome this potential issue, the interviewers stressed the importance of hearing what the participant thought about CHTC and that there were no wrong or right answers. However, participants may still have felt intimidated when talking to someone whom they may have assumed more “knowledgeable” than themselves about the issue.

## Results

### Overview of study participants

A total of 32 semi-structured interviews were completed with study participants including individuals who had ever been in an ongoing heterosexual relationship (*n* = 21) and key-informants (*n* = 11). Table 1 summarises demographic information for the 21 client participants (hereafter referred to as ‘individual participants’) by sex, age, relationship status, duration of the relationship, and HIV-testing history.

**Table 1:** Demographic and other information for individual participants (*N* = 21)

Individual participants	Female	Male
Age group		
< 35 years old	12	4
> 35 years old	3	2
Marital status		
Married (1st time)	11	3
Never married	3	1
Re-married	1	1
Co-habiting	0	1
Relationship duration		
< 5 years	9	4
> 5 years	6	2
HIV-testing		
Tested alone	4	3
Tested together with a partner	6	1
Tested alone and together with a partner	4	1
Never tested	1	1
HIV status		
HIV-positive	2	2
HIV-negative	3	1
Not disclosed	10	3

The 21 individual participants included 15 women and six men. More than one-third of participants reported being married. More than two-thirds had ever been tested for HIV, either as an individual or with a partner. Table 2 summarises information about the 11 key-informants (KI). The diversity of the sample in the current study as shown in Tables 1 and 2, helped to ensure a range of views were collected to inform the thematic analysis.

**Table 2:** Types of key-informants (*N* = 11)

Key-informants	N
Health care providers	
(Physician, VCT counsellor nurses, and PMTCT counsellor nurse/health officer)	5
Case-managers^a^	2
Religious leaders	4

^a^case manager are people who live with HIV and work as adherence counsellor at ART clinics in hospitals

### Key themes

**Fig. 1:**
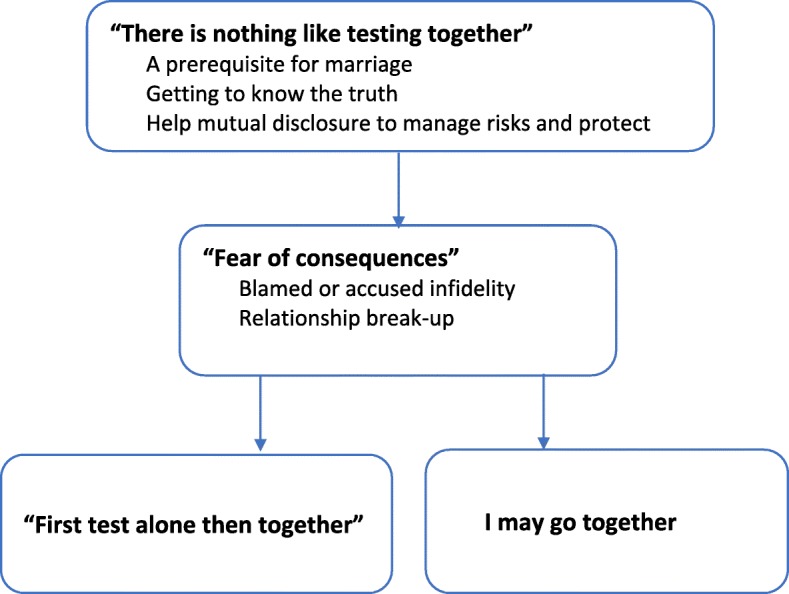
Conceptual model between the relationship social norm, consequences and HIV testing preference among people in a long-term heterosexual relationship

Participants voiced their perspectives on a range of issues related to Couples HIV Testing and Counselling (CHTC), and the results are reported under key themes as illustrated in Fig. 1. In general, many participants talked about the importance of undertaking HIV testing and counselling services with a sexual partner which is covered under the first major theme “there is nothing like testing together”. Key reasons offered by participants were that it was often considered a prerequisite for marriage by religious institutions or family, that it would help them to “know the truth” about their partner’s HIV status, and manage risks or protect their health if one of them is HIV positive. Across the data, the major second theme was “fear of consequences”. This fear was often then discussed by many participants as a reason why they would first think about going alone to check their HIV status before considering to undertake the test with a sexual partner as shown in the last section of Fig. 1. Most participants said they would first test alone before undertaking CHTC as represented by the larger box at the end of the figure, though it was possible some may undertake CHTC before first testing alone despite their fear of the consequences and this option is represented by a smaller box. The arrow with dashes between first testing alone and CHTC represents the possibility a person will undergo CHTC after first testing alone, though such a possibility appears to be remote if a person returns with an HIV positive result.

### There is nothing like testing together

When participants were first asked what they thought about CHTC they all said they knew what it was and in their initial response most highlighted the importance they saw in undertaking CHTC within an ongoing heterosexual relationship. The three main reasons given by participants for the belief that there was “nothing like testing together” were related to the ideals and requirements of marriage, finding out the truth, and managing risks or protecting health elaborated below.

#### Prerequisite for marriage

Most participants first expressed how vital undertaking couple’s HIV testing was by relating the act with the values of “marriage”:

*"In fact, there is nothing like to be tested [for HIV] with your partner. You see, it enables you to know what's in you and your partner. . . Why, because marriage is a shared life between the two people, it’s not just only one person” Female, 20’s-30’s (P-05)*

The notion of a “shared life” in marriage in the above quote suggests that there should be no secrets between the couple and this includes the couple’s previous sexual history and each persons’ HIV status. Religious leaders interviewed also often emphasised that couples should know each other and issues that matter most to build their partnership in the future echoing the comment of the individual participant above:

*"When we think about marriage, it’s an institution where two people decide to live a shared life as one. Therefore, we believe these two people should know each and everything about each other if they decided to live as one" Religious leader (KI-09)*

In Ethiopia, religious institutions that conduct marriage ceremonies, as well as parents of the bride and groom, may request evidence that a couple has had an HIV-test before proceeding with a ceremony:

*“Well, we [a religious institution] won’t be willing to undertake the [marriage] ceremony unless they [the couple] bring the [HIV test] result, because it’s apparent that they have responsibilities. You see, one of them could be sick [HIV-positive]. However, if they decided to carry on without having the test result, then we will not carry out the ceremony.” Religious leader (KI-09)*

It may be the case, as the above participant suggests, that the actual result is provided to the couples to show to the institution. Although a result for each person could be provided via CHTC, it is also possible that a test by each person undertaken independently of each other outside of CHTC could also be provided. What is not clear is whether a request for the actual result, regardless of testing type, is the norm. In the past, individuals who took the test used to be provided a certificate by the health facilities with the date of the test and the results for each person. This practice is now changing due to changes in policy directives:

*“Now a day, we don’t provide such evidence in the form of letter or certificate. Instead, we can tell the couple that if they want anyone whom they wished to know about the test results then they can bring the person with them to the testing centre, and we will let them know.” VCT counsellor (KI-02)*

It is unclear from the current study’s data whether this newer practice is occurring or not, but as with the provision of a certificate, it is enabling a third person to have access to the couple’s HIV test results, whether obtained together in CHTC or independently in individual testing. This has implications for individual privacy and rights. It is also possible that the serostatus of a couple may be considered by the religious institutions in deciding whether to mary the couple or not. However, having the test itself as a prerequisite to a marriage ceremony by a religious institution may not require the result itself to be disclosed, nor that each partner be HIV negative or sero-concordant. Interestingly, one religious leader was very clear that the couple’s serostatus did not matter and it was up to the couple to decide what they wanted to do:

*“As a [spiritual father], I can’t say ‘how do you love him/her? he/she is sick [HIV-positive]’. If I say: ‘how do you marry a person with HIV?’ Then why am I there? This means I have crossed the line. . . . If they don’t have a problem with being discordant, I will not have any problem with marrying them. This [HIV] is just a flesh’s sickness.” Religious leader (KI-07)*

In the above instance, the religious leader indicates he would respect the interest of individuals and enable them to make their own decision about marriage regardless of their HIV-serostatus. While this may not always be the case no-one spoke directly about interference by religious institutions in marriage decisions based on serostatus.

The other reason given by some participants to undertake CHTC was because of a request from parents of the bride and groom. In Ethiopia, there is a traditional process that will take place when two people decide to get married where the groom and his family will send elders (often very respected or well-known individuals) to request permission and blessings from the bride’s family. Nowadays this can also include the bride’s family wanting the couple to have HIV testing:

*“As you know in our culture, the groom’s family send elders to the bride’s family to request their approval and blessings in marriage. In this process, the parents and the elders would ask if the bride and groom are tested for HIV. Regardless of the groom’s personality or wealth, they need to see the test result, even in the most remote areas of the country. Otherwise, they won’t give their approval or blessings.” Female, 30’s-40’s (P-02)*

The family of the bride as revealed in this quote can sometimes seek to make sure that their daughter is in the right hands to have a stable marriage life and this now may include an HIV test result. Again the test result could be obtained through individual as well as CHTC. Regardless of the method, an HIV positive result for either partner may mean the marriage won’t proceed. One HIV-positive participant reflected that she thought it would be unlikely that the parents would give approval and blessings to proceed if the HIV test results were disclosed, and the couple was HIV-discordant (mixed HIV-status).

*“If she is my daughter, I will try everything to have a serious discussion with her…. I need to share what I know about HIV, or my personal experience so that they can understand what it means to be HIV-positive. I’m sure no one wants to play with fire with bare hands.” Case manager (KI-03)*

The notion of “playing with fire with bare hands” suggests that the idea of living with a sero-discordant partner is viewed by some as very dangerous and they may therefore interfere with the decision to marry. Again the lack of understanding of the treatments available to minmise the risk of transmission between partners is evident in the participants talk.

#### Getting to know the truth

The other aspect of CHTC that participants talked about was getting to know the truth. For some participants, CHTC was viewed as a means to get reliable information about their partner’s HIV-status if there is a lack of trust between the partners about their fidelity. Most of the participants said they would expect their partners to be truthful and wanted to have confidence in their partners in all domains of life including their HIV-status. However, some saw the process of taking the test together as a way to trust the information or test result. Participants’ comments also suggested that people may think that being asked to take the test together means their partner is suspicious of their sexual behaviour and commitment to monogamy:

*“It’s obvious; this [partner’s invitation to take HIV test as a couple] implies being suspicious.” Male, 31’s-40’s (P-11)*

In circumstances where partners don’t trust each other, it seems that CHTC could be initiated by one of the partners. This was evident in the excerpts below:

*“. . . it is good to go together if I’m suspicious that she is not going to tell me the truth about her status.” Male, 31’s-40’s (P-01)*

*“. . . if I don’t trust my partner, I wouldn’t take the test alone and allow my husband also to take the test alone at his convenient place. If I don’t trust him, I’ll bring him with me here [hospital] to take the test together . . .” Female, 20’s-30’s (P-06)*

Similarly, a senior Voluntary Counselling and Testing (VCT) worker commented on the trust issue from his experience at an HIV-testing clinic:

*“The other reason that makes a couple to decide to come here [testing centres] is due to trust issues. One of them, for instance, he may get suspicious that she [his partner] is going out with someone else likewise she may assume the same thing. In such circumstances, they challenge each other to check their [HIV] status and says, ‘why don’t we go together and check our [HIV] status?” VCT counsellor (KI-04)*

Challenging each other to undertake an HIV-test as a couple to prove or disprove a breach of trust, such as infidelity, is not the intention of CHTC and has implications for the relationship if the couple are found to be sero-discordant or both are found to be HIV positive. The faithfulness of a partner in the talk of these participants can be seen to merely rely on his/her HIV-status. Corroborating the sanctity of marriage in Ethiopia and the idea of fidelity, a religious leader expressed difficultly in the idea of suggesting HIV testing for those who are already married unless there are substantive reasons such as pregnancy or illness.

*"If we say go and take couples HIV testing, I think people could take it as we encourage or allow them to do something else – like encouraging or licencing the spouses to sleep with someone else than their marital partner. In fact, we don’t say like this or we have never said to anyone who is married to go and undertake the test and know your [HIV] status." Religious leader (KI-09)*

It appreared to be difficult for some of the religious leaders as revealed in the above quote to suggest couples who are in a marriage or relationship for quite some time to undertake CHTC. The suggestion could imply suspicion of unfaithfulness between partners. Nevertheless, the data strongly suggests that suspicion of infidelity is a key reason to initiate CHTC, and initiation of CHTC could be viewed as representing a lack of trust between the partners.

#### Helping mutual disclosure to manage risks and protect health

Some individual participants and key-informants talked about how CHTC could facilitate mutual disclosure of HIV-test results to manage risk of transmission by initiating medication as early as possible. Within an ongoing sexual relationship, disclosing one’s HIV-status to a partner could be a challenge for a person diagnosed HIV-positive. Two individual participants (a male who had never been tested for HIV, and a female who had taken HIV-testing with her partner) explained their views:

*“. . . if the wife or the husband came independently & found out that she/he is HIV-positive, it’s possible that both could fear to share the test results with their respective partners. It could be very difficult for the [HIV-positive] person to discuss about it with her/his partner. But, if you come together with your partner, you will get assistance from the [health] professionals.” Male, 20’s-30’s (P-04)*

*“I think the good thing of undertaking the [HIV] test together, it helps you to accept whatever the results are and continue living your life [the relationship]. Otherwise, you will end up living a life in which you struggle to hide things from each other which is very difficult.” Female, 20’s-30’s (P-06)*

In both participants’ excerpts, sharing individual HIV-positive status to a partner was seen as a difficult task that could be assisted by the CHTC process with professional help at the testing centres to understand and accept the results together and take the appropriate action, including medication and use of condoms. For example, an HIV-positive woman in thirty’s shared her view on how CHTC could help mutual disclosure to initiate ART as early as possible by comparing the stories of two HIV-positive women whom she met at a hospital while she was attending for ART.

*“I think a lot of people have benefited from this [undertaking CHTC] – for instance, I remember, one lady told me, just here in this hospital, that she was encouraged and accompanied by her husband. She said, ‘he saved my life on the day he brought me here to know myself [HIV-status] and start the medication’. In contrary, some individuals act differently. For instance, there was another woman I met in the same hospital while we were waiting. You know what happened? She told me her story with tears: she became paralysed because she didn’t start the medication [ART] early because she didn’t know that she was HIV positive. Whereas her husband was taking his medication [ART] without telling her that he is living with the virus.” Female, 31’s-40’s (P-02).*

In managing risks within an ongoing sexual relationship, many participants and key-informants also emphasised how undertaking CHTC could facilitate Prevention of Mother-to-Child Transmission (PMTCT).

*“Look, this [undertaking CHTC] is about life, it’s not something like some games. In addition, the child that I’m about to give birth should be free [HIV-negative] of the virus. We should do whatever it is necessary to prevent the occurrence of the infection in our child. Thus, it is a must. Female, 20’s-30’s (P-06)*

PMTCT, for most participants as noted in this excerpt, appeared to be an important issue and many participants expected parents to take responsibility to prevent the transmission to their unborn child by getting tested. For a pregnant woman who has been diagnosed HIV-positive, starting ART as soon as possible was seen as a priority to prevent the transmission of the virus to the unborn child. Yet, it could be a challenge for some women to take their medication home and start as prescribed by their physician due to fear of the consequences of their status becoming known to others, including their partner. For example, a key-informant who is HIV-positive and worked as an adherence counsellor highlighted how women’s economic power within a relationship could play a role in delaying the start of medication.

*“The women are afraid of telling their husband because in Ethiopia, many women are economically dependent on the men. The woman may be a housewife who doesn’t work. She will say “if I tell him the result, he will say you brought this [HIV] and you should leave”. Case manager (KI-08)*

In the talk of participants, the underlying assumption was that CHTC could make the process of learning about self and partner’s HIV status easier because the post-test counselling together afterwards could help both partners understand the results as well as promote initiation of medication and support for each other if one or both were HIV-positive. Healthcare providers in interviews also mentioned that they use CHTC as a strategy to help HIV-positive individuals including HIV-positive pregnant women who are afraid of disclosing their HIV-status to their partner, to disclose in a supportive environment:

*“. . . very often they afraid to tell . . . What we do is we tell the HIV-positive patient just to talk to their partner & say ‘a physician asked me to bring my partner to the clinic’. This will be without disclosing patient’s HIV test result. Then as soon as they arrive to the clinic, we’ll discuss with the partner who is not tested for HIV in the presence of their HIV-positive partner - your partner has been treated here just like any other patients [not related to HIV], but we haven’t seen any progress. Thus, we have decided to carry out HIV-testing, and we would like to encourage you both to take the test together.” Physician (KI-01)*

In Ethiopia, it is not known how widespread the practice discussed above, but such an approach has the potential to mitigate harms for those who may be vulnerable in trying to disclose without support from a health professional. It’s also important to take into account that a supportive environment and counselling at the health facilities during couple’s HIV-testing, and the extent and readiness of individuals to accept the HIV-test results are very important in considerating the utlity of CHTC services. However, despite a recurring reported view that CHTC is important within an ongoing sexual relationship, fear of the consequences was raised by many of the participants when talking about CHTC.

### Fear of consequences

Almost all individual participants stated that fear is what comes to their mind when considering taking an HIV test with a sexual partner. This fear was talked about in relation to what would happen following the test in the presence of their partner. Being diagnosed with the virus was imbued with a range of uncertainties about what would follow as one of the female participants who was HIV-positive clearly articulated:

*“It is fear. Regardless of your deeds, you’ll have this feeling inside of you, just fear. Even if you have nothing to worry about, but you just fear. I think that is the main reason [to people hesitate to undertake couple HIV testing]”. Female, 20’s-30’s (P-07)*

The notion of “your deeds” from this participant’s account suggests that the act of HIV testing was not merely considered as learning about one’s HIV serostatus; it’s also a process embedded with fear of its implications and consequences. As with the earlier sub-theme about getting to the truth, this participant’s talk suggests that even if she knows she has been faithful she is still afraid she may be HIV-positive and possibly accused of infidelity. Two dimensions of fear were found in the narratives of the participants: *fear of blame – an accusation of infidelity*; and *fear of break-up the relationship*.

#### Fear of blame or an accusation of infidelity

The first and most fundamental fear that participants raised across the interviews was fear of blame, referring to the concern of being judged or identified by others as having been unfaithful to a partner. As one participant said:

*“They [partners] may start saying that you are the one who brought this [HIV] into the relationship, no you’re the one. I think everyone knows what she/he has done in their life, but to protect yourself you may start blaming your partner.” Female, 20’s-30’s (P-08)*

This fear of being blamed is likely to be heightened if couples decide to take the test together, as they see that there is no way one can keep the information private. Thus, if there is an HIV-positive test result, either one or both partners may need to provide answers to questions about how this has happened:

*". . . soon after if they [couples] found out that one of them is [HIV] positive, they will ask ‘how this’s happened?’, ‘we were free [HIV negative] at the beginning [prior HIV-testing], but how this could happen?" Male, 20’s-30’s (P-04)*

*“. . . ‘where did you go? With whom have you slept?’ and then perhaps there will be fighting or quarrelling between the spouses.” Female, 20’s-30’s (P-06)*

In these excerpts, there appeared to be a common understanding that a person within an ongoing sexual relationship who is diagnosed HIV-positive would need to explain or justify their status to the HIV-negative partner. As explicitly spoken by one of the other participants “*.*. *. very often people fear how they can explain the result to their partner if it is HIV-positive*” *Female, 20’s-30’s (P-10).* Such expectations may put an HIV-positive person in a vulnerable position where they experience judgement of their fidelity even if they have remained faithful. The fear of breaking up from a partner was often discussed as putting the HIV-positive person in a vulnerable position of being judged and the assumption of infidelity.

*“You know why? It doesn’t give a nice impression if you separated from your partner and your partner tell everyone that the reason is you are HIV-positive. That doesn’t look good.” Male, 31’s-40’s (P-11)*

This participant was highly concerned that confidential information about an individual’s HIV-status could also be disclosed to other people without the consent of the person who is diagnosed HIV-positive. For example, he spoke about how partners who attended CHTC will know about their own and partner’s HIV-status, yet if the relationship is going to be terminated the confidential information could be shared more broadly in the community.

#### Fear of breaking-up the relationship

Fear of breaking-up, that is an anticipation of dissolving a marriage or partnership, was one of the recurring concerns that most male and female participants raised in relation to CHTC. If the test result turns out to be HIV-discordant, many of the participants presumed that was the end of the relationship:

*"Well, perhaps thinking about separating from your partner? Yeah, I think it is our worries that create the fear in our mind which is telling us that ‘you are going to separate if she is HIV-negative and you're [HIV] positive" Female, 20’s-30’s (P-07)*

*“. . . there is possibility of marriage dissolution, especially when there is a discordant result.” Male, 20’s-30’s (P-04)*

For some, the breaking up of their marriage which they have built over many years may be viewed as unbearable because the process may also involve and impact their children. Whereas for others, they said it would be unreasonable to maintain a relationship having a discordant HIV-status especially if the relationship is in its infancy. A male participant who is now single said:

*“Look if you take the test and know that you are HIV-positive while your partner is HIV-negative, then it’s meaningless to continue the relationship because you will hurt your partner.” Male, 31’s-40’s (P-11)*

A decision to break up a relationship here is to be made to protect a partner from becoming HIV-positive. Similarly, another participants also commented that it could be protecting self from being HIV-positive. This suggest that the process could hurt both HIV-positive and HIV negative partners. However, the likelihood of breaking up based on HIV-status varied from person to person. Some said they would take action straight away to dissolve the relationship: “*Well, you see if I’m infected with HIV and my husband is free [HIV-negative], or if I’m free [HIV-negative] and he is not, then we should be divorced because it’s a must*.” *Female, 20’s-30’s (P-06)*. Whereas, others like the following single female participant said the couple may find something that holds the relationship together regardless of being HIV-positive: “*The most important thing in a relationship is love; if there is real love, they will accept anything. So, the reason is they don’t have the true love for their partner*.” *Female, 20’s-30’s (P-10).*

The concern around CHTC was spoken about in terms of one’s partner knowing personal information about her/his partner’s HIV-serostatus, breaking up, others in society finding out this information, and what they may assume about the individuals involved. This meant that first testing alone was one major way that people would deal with the fears they expressed about CHTC.

### First test alone then together

There were a few participants who said they would like to go together with their sexual partners to take an HIV test and know about their partner’s HIV status directly from the health care providers. However, it was reported by most participants that they and people they knew would prefer to undertake an HIV test alone before taking the test together with their partner:

*“. . . most of the time people go to the testing centre alone to check their individual [HIV] status because of fear. It’s obvious you always think about your family and your community. You’ll never know what’s going to happen. Male, 51’s-60’s (P-03)*

*“I prefer to test alone because it’s better to know your own [HIV] status first. Then go together. For example, I would be very happy if I take the test alone, and after I know my result, then I’ll go with my husband. The only reason I say this is due to fear.” Female, 20’s-30’s (P-08)*

It was also evident here and among all the never married participants’ accounts that prior testing as an individual before undertaking the test with their sexual partner was preferred even if this meant the end of a relationship with no disclosure of their HIV status:

*“For me, I would prefer to take the test alone. Likewise, I expect that my partner would go alone. I don’t want to know that she is HIV-positive [if she is]. This means I would prefer separation without knowing that my partner is HIV-positive, and she doesn’t have to know that I’m HIV-positive [if I’m HIV-positive] either.” Male, 31’s-40’s (P-11).*

Despite a recurring view that CHTC was important in an ongoing sexual relationship in general terms, the participants in this study commonly reported taking or intending to take the HIV-test independently before undertaking the test together with their partner. This was also confirmed by the health care providers that often couples, especially those who are planning to get married, first they would go independently and then together.

## Discussion

The purpose of this study was to understand how men and women who had ever been or were currently in an ongoing heterosexual relationship in Ethiopia understood and intended to use CHTC. The findings suggest that participants recognised CHTC as one of several HIV-testing and counselling approaches and expressed some perceived benefits of this testing method for couples, including to know “the truth” about a partner’s HIV status and prevent HIV infection. However, the study findings show that some couples may not use CHTC services if an individual believes it will expose them to negative consequences if they are found to be HIV positive, such as judgments about their faithfulness, breaking up of the relationship and their status becoming known by others in the community. As a way of overcoming the fear of potential consequences, participants described a way to use available HIV-testing services that worked for them and which reduced the likelihood of negative consequences - first test alone and if HIV-negative then test together with a sexual partner.

This is not the first-time people and the communities they belong to have devised their own strategies to prevent and control HIV-transmission by subverting or modifying expected health care system approaches. The “talk, test, test, trust” strategy among gay men in Sydney is an example where gay men developed a ‘negotiated safety’ strategy which helped them sustain safe sexual practices [34, 35]. This strategy was a community driven and grass-roots response to addressing safe sex in their relationships with the wider availability of HIV testing in Australia. After the strategy was identified by researchers as already occurring in the community, health care professionals advocated and promoted it. In the current study, although the health care system and national HIV policy in Ethiopia encourages couples to attend HIV testing simultaneously, the study findings suggest that ‘first testing alone’ may be a strategy employed by at least some heterosexual couples before CHTC and sometimes instead of CHTC. This seems a reasonable strategy for individuals given the consequences feared by participants in the Ethiopian context.

Participants in this study consistently expressed their fear of consequences following CHTC. The most significant anticipated consequence included: blame for being unfaithful by their partner and in society more generally and breaking-up of the relationship. These fears have also been found in other studies [14–16] in Uganda, where suspicion of infidelity was a key reason for some individuals in a relationship to initiate CHTC. However, positioning of CHTC in the community as a way to uncover infidelity is fraught. For example, it becomes a process that can undermine trust between partners and constitutes a threat to the relationship if one of the partners is found to be HIV-positive. This fear of being blamed and accused of infidelity is further exacerbated by the possibility that a third person or party (religious leaders and parents) may have access to an individual’s test result as evident in the views of participants in the current study. These findings lend support to those of another qualitative study in Zambia [36] which also found couples agreed that CHTC could provide opportunities to facilitate disclosure, but many saw CHTC as a process imposed by people with considerable power in the community, such as health care providers, religious leaders and parents [14]. The current study supports the notion that these social and institutional pressures can weigh heavily in a couple’s decision to undertake CHTC undermining their sense of having any rights or power in the decision and control of who may have access to their test results.

It is noteworthy that in the current study many participants did initially express a positive view of having an HIV test with a sexual partner. The theme “there is nothing like testing together” reflects this view. However, this view may be at least partly driven by social norms fueled by the requirements of religious institutions and parents before getting married alongside their view that CHTC is a good way to “find out the truth” when they suspect infidelity. Along with an emerging body of evidence [14–16, 19], the findings in this study also showed that there may be potential harms in promoting CHTC for all couples as an HIV testing approach in countries where stigma and misunderstanding about the disease, its transmission and treatment remain a problem [37, 38] and where CHTC is seen as a key way to prove infidelity. Thus, individuals may employ an approach that minimises risk to themselves by choosing to first go alone to have an HIV test before undertaking CHTC.

The findings in this study confirm that many people are fearful of having an HIV test that may place them in a situation where, without their proper consideration and choice, an HIV positive test result and their HIV status is communicated directly or indirectly to others. People are rightfully concerned about the implications of being identified as living with HIV and the stigma and discrimination that may come with that status. Having an HIV-positive test result used as way of proving infidelity was a major concern of participants. And such concern has the potential to jeopardize public health efforts to increase the uptake of HIV testing among people in ongoing sexual relationships. Though participants in this study initially indicated some positive sentiment about CHTC, a recent systematic review and meta-analysis supports the current study findings that such positive overall sentiment does not necessarily translate to practice, with nearly three out of four couples in SSA countries choosing not to use CHTC as their first HIV testing option [39]. Together these findings underscore the need to offer a range of testing options to couples in ongoing sexual relationships. Further research is needed to better understand the harms and potential benefits of CHTC in SSA countries, particularly hearing from those who have experienced having an HIV test through CHTC. A quantitative study design is recommended to test the generalizability of the findings from this qualitative study.

### Strengths and limitations of the study

The findings of this study add to the scientific knowledge on individual perspectives, concerns and intentions to undertake HIV-testing while in an ongoing heterosexual relationship. The findings highlight the interconnection between individual concerns and common practices of HIV-testing within an ongoing sexual relationship. The study participants were sufficient in number and diverse in age, gender, relationship status, HIV-testing experiences and HIV-status to answer the research question.

The study has some limitations. As a qualitative study, the findings are not representative and ought to be considered within the context of the study. A majority of the participants were interviewed within the health facilities. Doing so may have influenced participants directly or indirectly to provide socially desirable responses, in line with more positive sentiments about CHTC services provided by the health facility in which they were being interviewed. Such potential influences were anticipated by the study team and steps were taken to try and avoid biasing the data. For example, participants were informed that the study team was independent of the health service and that data would not be shared with the health service and that data never be reported in a way that could identify any individual. Despite these steps, participants may have been reluctant to criticise the services.

## Conclusion

Individuals who were in an ongoing heterosexual relationship in this study perceived CHTC as an important HIV testing approach, mainly for the purpose of future planning of marriage, minimising HIV-transmission risks and as an attempt to verify any breaches of trust. Participants also had serious reservations about undertaking an HIV test with a sexual partner. To overcome such fear and to avoid anticipated consequences of blame for infidelity, break-up of a relationship, and others finding out their status, many individuals preferred to use individual-based HIV-testing services prior to CHTC services. There is a need for quantitative research to examine how widespread these views and practices are in the community. Further research also needs to examine people’s direct experience of CHTC to better understand how this approach impacts individuals and how it may be more effectively implemented to minimise any potential harms.

## Supplementary information

**Additional file 1.** Interview topic guide for Key-informants.

**Additional file 2.** Interview topic guide for individual participants.

## Data Availability

All data on which the results and discussions for this paper are based is available from the corresponding author upon reasonable request.

## Abbreviations

ANC: Antenatal Care
ART: Antiretroviral therapy
CHTC: Couples HIV Testing and Counselling
HTC: HIV Testing and Counselling
KI: Key Informant
PLHIV: People Living with HIV
PMTCT: Prevention Mother to Child Transmission
RA: Research assistant
SSA: Sub-Saharan Africa
TB: Tuberculosis
UNSW: University of New South Wales
VCT: Voluntary Counselling and Testing
WHO: World Health Organization
