# Supplementary material for: Beliefs and intention of heterosexual couples about undertaking Couple’s HIV Testing and Counselling (CHTC) services in Ethiopia
Source: BMC Health Serv Res. 2020 Feb 5;20:92. doi: 10.1186/s12913-020-4947-7 (PMC7003442; doi:10.1186/s12913-020-4947-7)
Supplement: Supplementary file 1 — Additional file 1. Interview topic guide for Key-informants. [file 12913_2020_4947_MOESM1_ESM.docx]

# Interview topic guide for Key-informants

1. Introduction
   1. Thank you for your time and interest in taking part in this study to share your view. Before we start with specific questions, please tell us about you; your role & responsibilities in this institution?
   2. How long have you been working/serving?
   3. Good, let me move to the questions. In this institution, I was wondering what kind of preparation, teaching or counselling you are providing for couples during a pre-marital stage or after they got married related to HIV testing.

Probes:

- Ask reasons for services or expectations these institutions have regarding HIV testing services
- Ask what these institutions will do if the test results become HIV-positive for one or both partners.

1. **Intention**
   1. Overall, how do you see the current intention to undertake CHTC among couples? What would be the reasons for such a trend of intention?
2. **Perceptions**
   1. What do you think are the benefits or advantages of undertaking HIV testing as a couple?
   2. On the contrary, what do you think are the disadvantages or risks of undertaking the test together as a couple?
3. **Social norms and factors**
   1. OK, let me move on to another issue. Among different kinds of people found around the couples, who do you think encourages or approves the individual’s intention to undertake HIV testing with their partner? Why?
   2. On the contrary – whom do you think that discourage or disapprove the individual’s intention to undertake the test together with their partner? Why?
   3. Whose opinions about CHTC do you think are important to these couples? Why?
   4. What do you think are enablers which make undertaking couples HIV testing for individuals who are in an ongoing relationship easy? Why?
   5. What factors or circumstances would make it easier for individuals to go with their partners for HIV testing and counselling services? Why?
   6. What factors or circumstances would make it difficult or impossible for individuals to go with their partners for HIV testing and counselling services? Why?
   7. You can share your view on the following question based on your personal and work experiences or observation. Comparing the two testing approaches – undertaking the test separately as an individual vs couples testing, which one do you think individuals most prefer? Why?
4. **Closing**
   1. Thank you for your time and valuable information. Before we closing our discussion, is there anything else I haven’t asked you that you wish to add?
