# Supplementary material for: Beliefs and intention of heterosexual couples about undertaking Couple’s HIV Testing and Counselling (CHTC) services in Ethiopia
Source: BMC Health Serv Res. 2020 Feb 5;20:92. doi: 10.1186/s12913-020-4947-7 (PMC7003442; doi:10.1186/s12913-020-4947-7)
Supplement: Supplementary file 2 — Additional file 2. Interview topic guide for individual participants. [file 12913_2020_4947_MOESM2_ESM.docx]

# Interview topic guide for individual participants

1. Introduction

Thank you for your interest in taking part in this study. Let’s start with some introductory question:

- 1. How was your day?
  2. May I ask your age?
  3. Would you like to share your relationship story? How did you meet with your partner?

Probe:

- Duration of the current/last relationship? How long have you been together?
- Are you married?
- Any children?

1. **Intention**
   1. What do you know about Couples HIV Testing and Counselling (CHTC)? (if no little, will briefly explain)
   2. How likely do you think couples are to go together for HIV testing and counselling?

Probes: why do you think that?

- 1. Have you ever been tested for HIV?

Probes: If yes, was it alone or with your sexual partner?

- 1. If the person responds Q. No to 2.3. probe - have you thought about going for HIV testing with your partner?

Probes: If yes/no, Why? Tell more about it

1. **Perceptions**
   1. What do you feel about CHTC?

What is good about CHTC?

What do you think are possible problems with CHTC?

- 1. What do you believe are the advantages of couples taking HIV testing and counselling services together?
  2. What do you believe are the disadvantages of couples taking HIV testing and counselling services together?
  3. What do you believe are the benefits of couples taking HIV testing and counselling services together?
  4. What do you believe are the risks of couples taking HIV testing and counselling services together?

1. **Social norms and factors**
   1. Are there any individuals or groups who would approve of your intention/decision to attend for CHTC? Why do you think they approve of your intention?
   2. Are there any individuals or groups who would disapprove of your intention/decision to attend for CHTC? Why do you think they disapprove of your intention?
   3. Whose opinions about CHTC are important to you?
   4. Would there be anything else important in your intention/decision to CHTC?
   5. What factors or circumstances would make it easier for you to go with your partner for HIV testing and counselling services?
   6. What factors or circumstances would make it difficult or impossible for you to go with your partner for HIV testing and counselling services?
   7. How likely do you think CHTC is to be acceptable among other couples?

Why do you think the reasons are?

- 1. What do you think are the most important factors that may make CHTC acceptable for you or others you know?
  2. What do you think are the most important factors that may make CHTC not acceptable for you and others you know?

1. Closing
   1. Thank you for your time and valuable information. Before we are closing our discussion, is there anything else I haven’t asked you that you wish to add?
